# Tularemia Transmission to Humans, the Netherlands, 2011–2021

**DOI:** 10.3201/eid2804.211913

**Published:** 2022-04

**Authors:** Jolianne M. Rijks, Anna D. Tulen, Daan W. Notermans, Frans A.G. Reubsaet, Maaike C. de Vries, Miriam G.J. Koene, Corien M. Swaan, Miriam Maas

**Affiliations:** Dutch Wildlife Health Centre, Utrecht University, Utrecht, the Netherlands (J.M. Rijks);; European Programme for Intervention Epidemiology Training (EPIET), European Centre for Disease Prevention and Control, Stockholm, Sweden (A.D. Tulen);; National Institute for Public Health and the Environment, Bilthoven, the Netherlands (A.D. Tulen, D.W. Notermans, F.A.G. Reubsaet, M.C. de Vries, C.M. Swaan, M. Maas);; Wageningen Biodiversity Research, Wageningen University and Research, Lelystad, the Netherlands (M.G.J. Koene)

**Keywords:** tularemia, *Francisella tularensis*, bacteria, zoonoses, human, transmission, ecology, the Netherlands

## Abstract

We used national registry data on human cases of *Francisella tularensis* subspecies *holarctica* infection to assess transmission modes among all 26 autochthonous cases in the Netherlands since 2011. The results indicate predominance of terrestrial over aquatic animal transmission sources. We recommend targeting disease-risk communication toward hunters, recreationists, and outdoor professionals.

*Francisella tularensis* subspecies *holarctica* bacteria are the main causative agent of tularemia in Europe (*1*). The pathogen can be transmitted to humans from animals, vectors, food and water, or the environment, through broken skin or via conjunctival, oral, or respiratory routes. The clinical manifestation of tularemia in humans can be ulceroglandular, glandular, oculoglandular, oropharyngeal, pneumonic, or typhoidal. The bacterium has a complex ecology and 2 interconnected lifecycles: a terrestrial lifecycle associated primarily with lagomorphs, small rodents, ticks, and tabanids; and an aquatic lifecycle associated with mosquitoes, semiaquatic animals such as beavers, contaminated water, and mud (*1*). The relative contribution of these lifecycles to human tularemia varies among countries (*1*).

In the Netherlands, no autochthonous human cases were reported during 1953–2010 (*2*), even though notification was mandatory during January 1976–April 1999. However, since 2011, multiple autochthonous human tularemia cases caused by *F. tularensis* subsp. *holarctica* infection have been detected (*2*–*5*) and systematically registered; mandatory notification was reinstated in 2016. In addition, the bacterium has been detected since 2013 in European brown hares (*Lepus europaeus*) and Eurasian beavers (*Castor fiber*), as well as in surface water (*6*–*8*). No wildlife cases were reported during 1953–2012 (*6*). 

To target preventive measures and communication regarding human tularemia requires insight into the main transmission modes and identification of the lifecycle. We assessed the distribution of transmission modes in autochthonous human tularemia cases in the Netherlands, using the national registry of tularemia cases. We extracted data from all autochthonous human cases from 2011–2021 from the National Public Health Institute database. Public health authorities identified the most probable transmission mode of each case at the time of diagnosis on the basis of the clinical presentation of the disease combined with information on exposure, either occupational or nonoccupational, obtained from standardized interviews with each patient (https://lci.rivm.nl/sites/default/files/2018-10/LCI-richtlijn%20Tularemie%20-%20bijlage%203%20vragenlijst%20Osiris.pdf). We aggregated cases per transmission mode and allocated them to either the terrestrial or aquatic lifecycle of *F. tularensis* subsp. *holarctica*. We considered the transmission mode confirmed if the source was an animal carcass that tested positive for *F. tularensis* subsp. *holarctica* by quantitative PCR or culture; otherwise, the mode remained probable. We included clinical manifestations and basal clade data of cases in the overview if available.

In total, we analyzed 26 human cases from across the country, all but 2 in male patients. Median age was 52 (range 1–78) years. In 23 cases, the source was confirmed (n = 2) or probable (n = 21). Of these, 16 cases were allocated to the terrestrial lifecycle, and 7 to the aquatic lifecycle. In 3 cases, the transmission mode was unclear; we excluded these cases from further analysis (Table). 

**Table:** Overview of autochthonous human tularemia infections reported in the Netherlands, 2011–2021

Life cycle	Transmission mode	Probable or confirmed mode	Year	Occupational exposure	Clinical manifestation	Basal clade	Reference
Terrestrial	Aerosols from contaminated vegetation	Probable	2016	Yes	Pneumonic	B.6-B.11	(*4*)
		Probable	2017	Yes	Pneumonic	ND	
	Contact with (or consumption of) infected hare carcass	Probable	2014	No	Ulceroglandular	ND	(*3*)
		Probable	2014	No	Ulceroglandular	ND	(*3*)
		Confirmed	2014	No	Glandular	B.12-B.20	
		Confirmed	2016	No	Ulceroglandular	ND	
		Probable	2016	No	Ulceroglandular	B.6-B.11	
		Probable	2017	No	Unclear (fatigue)	ND	
		Probable	2019	No	Oculoglandular and oropharyngeal	ND	
		Probable	2021	No	Glandular	ND	
	Mouse bite	Probable	2021	No	Ulceroglandular	B.6-B.11	
	Tick bite	Probable	2019	No	Glandular	ND	
		Probable	2020	No	Glandular	B.12-B.33	
	Insect bite while on land	Probable	2013	No	Ulceroglandular	B.6-B.11	
		Probable†	2016	No	Glandular	B.12-B.33	
		Probable	2021	Yes	Ulceroglandular	B.6-B.11	
Aquatic	Contact with contaminated water/mud	Probable†	2016	No	Glandular	ND	(*5*)
		Probable	2016	Yes	Ulceroglandular	ND	
		Probable	2016	No	Glandular	ND	
		Probable	2016	No	Ulceroglandular	ND	
	Contact with contaminated water or insect bite	Probable†	2015	No	Ulceroglandular	B.6-B.10	
		Probable	2021	No	Oculoglandular and ulceroglandular	ND	
	Insect bite while on water	Probable	2011	No	Ulceroglandular	B.6-B.11	(*2*)
Unclear	Unclear	Probable	2016	No	Glandular	ND	
		Probable	2018	No	Ulceroglandular	B.12	
		Probable	2018	No	Glandular	B.6	

*Data are for 26 infections caused by *Francisella tularensis* subspecies *holarctica.* ND, not determined. †Water, sampled within 6 weeks from waterbodies in the area where infection was assumed to have occurred, tested positive for *F. tularensis* subsp. *holarctica* by quantitative PCR, indicating presence of the bacterium in the local environment around the time of infection and highlighting the interconnection between lifecycles (*7*).

Occupational exposure was likely in 4/23 cases: 1 case-patient was probably infected while tending to cattle in pasture, the other 3 while performing vegetation maintenance, and 2 of those 3 had the pneumonic tularemia, which was reported in no other patients (Table). The strain from 1 pneumonic case-patient had been characterized previously as belonging to basal clade B6 (*4*), supporting previous associations found between pneumonia and basal clade B6 in both humans and hares (*8*,*9*).

Nonoccupational exposure through contact with infected terrestrial mammals was likely in 9 cases. Of those, 8 were assumed or confirmed to be infected by contact with infected hares, mainly through skinning and rarely through consumption. These case-patients were mostly hunters (n = 7) who showed diverse clinical symptoms; 2 cases were related to the same hare (Table). The ninth case concerned an ulceroglandular infection from a mouse bite (Table), a mode previously described in Switzerland (*10*). Nonoccupational exposure through arthropod bites, contaminated water, or mud was likely in the remaining 10 case-patients, who contracted tularemia while performing recreational outdoor activities in a terrestrial (4/10) or aquatic environment (6/10) (Table).

These results support the need for ongoing tularemia risk and prevention communication to hunters, and they identify a need for communication to outdoor (water) recreationists and to professionals such as grounds maintenance workers and foresters. Physicians must be aware of these risk groups and the diversity of clinical presentations for early identification and treatment.

The relative importance of the terrestrial lifecycle as a source of human infections in the Netherlands is consistent with the rare and sporadic occurrence of cases; human tularemia cases from aquatic sources are more likely to occur as large outbreaks (*1*). Nevertheless, local disease ecology can change over time, and the Netherlands is a low-lying, water-rich country in which favorable conditions for *F. tularensis,* such as floodplains and meandering waterways, are promoted to buffer excess rainfall due to climate change. It is therefore relevant to continue monitoring the transmission routes in human tularemia cases for early detection of shifts in tularemia lifecycle contributions, which may require adaptation of risk and prevention communications.
